# Feasibility and efficacy of neck dissections in patients with thyroid cancer

**DOI:** 10.1186/1756-6614-6-S2-A63

**Published:** 2013-04-05

**Authors:** Małgorzata Wierzbicka, Edyta Gurgul, Elżbieta Waśniewska-Okupniak, Maria Gryczyńska, Tomasz Piorunek, Marek Ruchała

**Affiliations:** 1 Department of Otorhinolaryngology, Head and Neck Surgery, Poznan University of Medical Sciences, Poznan, Poland; 2 Department of Endocrinology, Metabolism and Internal Diseases, Poznan University of Medical Sciences, Poznan, Poland; 3 Department of Pulmonology, Allergology and Respiratory Oncology, Poznan University of Medical Sciences, Poznan, Poland

## Introduction

The aim of the study was to assess (1) the feasibility and the outcomes of secondary neck dissections in well differentiated, medullary and poorly differentiated thyroid cancer and (2) to evaluate complications associated with surgical treatment.

## Material and methods

We assessed the results of secondary neck dissections in 51 patients previously operated for thyroid cancer: 33 well differentiated thyroid cancer, 15 medullary cancer, 3 poorly differentiated thyroid cancer.

## Results

Reoperations covered I-VII neck levels. Radical neck dissection was performed in 20 patients, selective neck dissection (SND) in 31 patients. These included 16 central compartment (CC) and 10 mediastinal excisions. Postoperative complications were stated in 13 patients: 4 chyle leaks, 3 massive bleedings, 8 injuries of RLN, hypoparathyroidism in 22 patients, 2 patients died in perioperative period. Stimulated Tg<2mg/ml was observed in 7 patients with WDTC during the first control after neck dissection; in 6 patients Tg level decreased after operation; 7 patients had still notably elevated Tg levels (>30 ng/ml). None of the patients with medullary cancer achieved calcitonin level lower than 10 pg/ml; 9 patients developed distant metastases.

## Conclusions

Patients with nodal metastases deriving from thyroid cancer present a challenging group for surgeons. The policy is to operate due to strong indications. It is important to be aware of possible complications. The outcomes of neck dissections in patients with medullary and poorly differentiated thyroid cancer were unsatisfactory.

